# Supplementary material for: Mechanical Robust, Self-Healable Polyurethane Elastomer Enabled by Hierarchical Hydrogen Bonds and Disulfide Bonds
Source: Polymers (Basel). 2023 Oct 8;15(19):4020. doi: 10.3390/polym15194020 (PMC10575067; doi:10.3390/polym15194020)
Supplement: Supplementary file 1 [file polymers-15-04020-s001.zip › polymers-2624371-supplementary.pdf]

Supporting information

Mechanical Robust, Self-Healable Polyurethane Elastomer Enabled by Hierarchical Hydrogen Bonds and Disulfide Bonds

Biqiang Jin [1,2,*], Wenqiang Wu [3,*] and Haitao Wu [2,*]

[1] College of Science, Xichang University, Xichang 615000, China
[2] State Key Laboratory of Polymer Materials Engineering, College of Polymer Science and Engineering, Sichuan University, Chengdu 610065, China
[3] Sichuan Dowhon New Material Co.ltd, Meishan 611734, China
* Correspondence: elden_jin@163.com (B.J.); wwqatl@126.com (W.W.); wht2281007108@163.com (H.W.)

Table S1. Composition and feed ratio of the fabricated PU elastomer.

Sample	PTMEG (2000 g/mol)	IPDI	DBTDL	Chain extender	DMF
PU	0.0075 mol (15 g)	0.015 mol (3.33 g)	0.1 ml	0.0075 mol (1.86 g)	50 ml

Table S2. Summary of mechanical properties of the obtained PU elastomer.

Sample	Mechanical strength (MPa)	Strain at break (%)	Toughness (MJ/m^3)
PU	9.9 ± 4.5	1427.8 ± 15.3	44.87 ± 6.9

Table S3. Summary of self-repair efficiency defined in terms of elongation at break.

Healing conditions	Self-healing efficiency
25 °C healing 12 h	81.0%
25 °C healing 24 h	86.6%
40 °C healing 12 h	114.3%
60 °C healing 12 h	102.1%

Note: The self-repair efficiency, defined as elongation at break, is over 100%, which may seem strange but is understandable. This is due to the fact that the molecular chains at the fracture surface are better recombined and the network is more complete, so that the repaired specimens undergo tensile testing at a different location to where the original damage occurred, resulting in a higher elongation at break and therefore a much higher self-repair efficiency.
